# HTLV-1-infected HIS Rag2-/-γc-/- mice, a suitable model for in vivo investigating the effects of drugs in ATL treatment?

**DOI:** 10.1186/1742-4690-8-S1-A11

**Published:** 2011-06-06

**Authors:** Julien Villaudy, Anne Cachat, Sandrine Alais, Hiba El Hajj, Louis Gazzolo, Ghazi Zaatari, Olivier Hermine, Hughes de Thé, Ali Bazerbachi, Madeleine Duc Dodon

**Affiliations:** 1 Virologie Humaine, INSERM U758, Ecole Normale Supérieure de Lyon, IFR 128 BioSciences Lyon-Gerland, Lyon, 69364 Cedex 07, France; 2 Department of Internal Medicine, American University of Beirut, Beirut, Lebanon; 3 Department of Pathology and laboratory Medicine, American University of Beirut, Beirut, Lebanon; 4 CNRS UMR8603, Hôpital Necker, Paris, 75743 Cedex 15, France; 5 Université Diderot-Paris, INSERM U728, Hôpital Saint-Louis, Paris, 75475 Cedex 10, France

## 

Adult T cell Leukemia, an aggressive T-cell malignancy linked to HTLV-1 infection, is resistant to chemotherapy. Recently, promising results were obtained with the combination of arsenic trioxide, interferon alpha and zidovudine. However, the cellular and molecular mechanisms of their anti-leukemia activity remain to be investigated. To that aim, we have relied on HIS (Human Immune System) Rag2-/-γc-/- mice. We have indeed observed that when infected with HTLV-1, these mice displayed, five months later, an elevated number of human Tax-expressing T cells in the thymus, the spleen and the mesenteric lymph nodes. Some of them also developed T lymphoproliferative diseases.

To determine the effects of these three drugs on the apparition of these pathological features, Rag2-/-γc-/- mice were infected with HTLV-1 and, 16 weeks after infection, daily treated with the drugs for one week and sacrificed. Untreated HIS mice infected with HTLV-1 were used as control. Treatment resulted in a significant decrease in the spleen weight as compared to untreated controls. Interestingly, we also noted a decrease of the proviral load in thymocytes as well as a drop in the number of mature activated T cells in the spleen and in lymph nodes. These data clearly indicate that the HIS mouse model provides us with the opportunity not only for the pre-clinical evaluation of therapeutic approaches against the leukemogenic process associated with HTLV-1 infection, but also for unraveling the corresponding mechanisms.

